# An Appraisal of Narcissistic Rage Through Path Modeling

**DOI:** 10.1177/08862605221084746

**Published:** 2022-03-25

**Authors:** David Théberge, Dominick Gamache

**Affiliations:** 1 Department of Psychology, 14847 Université du Québec à Trois-Rivières, QC, Canada; 2 CERVO Brain Research Centre, Université Laval, Quebec City, QC, Canada

**Keywords:** narcissistic rage, narcissistic vulnerability, narcissistic grandiosity, borderline traits, shame, aggression, path analysis

## Abstract

Pathological narcissism and borderline traits have been consistently associated with interpersonal aggression. Shame has been identified as an important trigger of aggressive behaviors in individuals with pathological personality traits, especially for narcissistic vulnerability and borderline traits. This is in line with Kohut’s theory on narcissistic rage, that is, aggression, anger, and destruction that act as a protection for a grandiose self. The present study aims to investigate the interrelations between pathological narcissism, borderline traits, shame, and trait aggression, concepts that are parts of the narcissistic rage phenomenon introduced by Kohut, using path models. A total of 399 participants completed self-report questionnaires assessing personality traits (narcissistic grandiosity and vulnerability, and borderline traits), shame, and aggression. Three path models including these variables were tested and compared to one another on fit indices. Results show that shame acts as a mediator between pathological traits (narcissistic vulnerability and borderline traits) and trait aggression, whereas the relationship between narcissistic grandiosity and aggression was direct (i.e., shame was not involved). Results expand the narcissistic rage theory by suggesting that it might represent an internalizing type of aggression that manifests in the context of narcissistic vulnerability and borderline traits, which is not the case for narcissistic grandiosity that exerts a direct effect on trait aggression.

## Introduction

Violent behaviors are overrepresented in the media, and are often depicted as random, unforeseeable, and meaningless, especially when they are perpetrated by someone with a psychopathology (Stuart, 2003). A systematic literature review by Yu et al. (2012) explored the relationship between personality disorders (PD) and antisocial behaviors, including aggression. They found that the presence of any PD was associated with a threefold increase in the risk of perpetrating antisocial behaviors compared with the general population. In other studies pertaining to specific PDs, pathological narcissism and narcissistic PD (e.g., Rasmussen, 2016; Vize et al., 2019), as well as borderline personality disorder (BPD; e.g., Mancke et al., 2015; Peters & Geiger, 2016), have been consistently linked to aggression.

### Pathological Personality Traits and Aggression

#### Pathological narcissism.

The aggressive component of narcissism has been abundantly described in the psychoanalytic literature, although authors do not always agree on whether aggression should be seen as primary (e.g., Kernberg, 1984) or secondary (i.e., as an understandable response to parental failures; Kohut, 1971). Another debate regarding the conceptualization of pathological narcissism pertains to the existence of more than one phenotype. The *Diagnostic and Statistical Manual of Mental Disorders* (American Psychiatric Association, 2013) Section II operationalization has garnered mitigated reactions as the focus of the diagnostic criterion is mostly on grandiose aspects and feelings of entitlement, while omitting a vulnerable side to pathological narcissism observed in clients (Pincus et al., 2014). Nowadays, contemporary authors from different backgrounds (e.g., Tanzilli & Gualco, 2020; Weiss & Miller, 2018; Wright & Edershile, 2018) endorse a multidimensional conceptualization of pathological narcissism including a grandiose and a vulnerable presentation. Narcissistic grandiosity pertains to an inflated self-image, feelings of entitlement, arrogance, risk taking, and antagonism in interpersonal relationships (Miller et al., 2021). On the other end, narcissistic vulnerability is characterized by feelings of despair, emptiness, and shame, with underlying grandiose fantasies (Kealy & Rasmussen, 2012; Weiss & Miller, 2018).

In a recent study using dominance analysis to examine the relationship between narcissism and aggression, both phenotypes have been significantly related to aggression; narcissistic grandiosity was more strongly associated with proactive aggression, whereas narcissistic vulnerability was better characterized by reactive aggression (Vize et al., 2019). In another research, Hart et al. (2017) studied aggressive responses to provocation in both narcissistic grandiosity and vulnerability using an experimental paradigm. Results show that narcissistic grandiosity was associated with aggression and hostile behaviors, but its association with emotional responses was more ambiguous; in fact, it was negatively correlated with sadness and other painful feelings, and uncorrelated with anger. Narcissistic vulnerability was also associated with aggression and hostile behaviors but, contrary to narcissistic grandiosity, it was associated with a significant increase in negative emotions as well.

#### Borderline pathology

The clinical picture of borderline pathology includes not only emotional dysregulation and suicidal behaviors but also impulsive aggressive behaviors (Bohus et al., 2021). Emotional dysregulation and a tendency to misinterpret facial expressions as threats have been pointed out by Mancke et al. (2015) as predispositions for BPD individuals to reactive aggression, anger, and hostility. Moreover, structural neuro-imagery studies revealed smaller amygdala and hippocampus, and abnormalities in gray matter volume in prefrontal and limbic cortex of individuals with BPD—which are all structures involved in aggressive behaviors, affective dysregulation, and impulsivity (Mancke et al., 2015). A correlational study (Peters & Geiger, 2016) also positively linked BPD with aggression, anger, and hostility. This same study also revealed that shame could fuel hostility and aggression within these individuals.

### Shame: A Pathway to Aggression

The General Model of Aggression (Anderson & Bushman, 2002) posits that the interaction between a person and a situation may generate affects, cognitions, and a physical arousal that may result in impulsive actions (e.g., aggression) or in thoughtful outcomes. For example, shame—a particularly painful emotion that can be summarized as a global, negative self-evaluation following the exposure of a part of ourselves we wanted to remain concealed (Dearing & Tangney, 2011; Nathanson, 1992)—could trigger impulsive aggression in a given person in a certain situation.

The role of shame and threatened egotism^1^ in interpersonal aggression has been extensively studied (e.g., Bushman & Baumeister, 1998; Elison et al., 2014; Velotti et al., 2017). Previous research shows positive associations between shame and aggression, and with pathological narcissism and borderline traits as well (Schoenleber & Berenbaum, 2012). Indeed, narcissistic vulnerability was tied to shame feelings in theoretical (e.g., Kealy & Rasmussen, 2012) and empirical (e.g., Pincus et al., 2009; Poless et al., 2018) writings. The associations between narcissistic grandiosity and shame are, however, more controversial; in some studies (e.g., Poless et al., 2018), narcissistic grandiosity was negatively correlated with shame, whereas other studies (e.g., Pincus et al., 2009; Théberge et al., 2021) report positive—albeit weak to moderate—correlations between the two. Negative correlations reported in the literature between narcissistic grandiosity and shame are aligned with the theoretical assumption that grandiosity acts as a defense against shame feelings (Tracy et al., 2011), and it seems that the valence of the correlations could be contingent upon the chosen measure of narcissism (Di Sarno et al., 2020).

On the other hand, shame is also a central feature in borderline pathology, although it does not stand as a diagnostic criterion for BPD (Buchman-Wildbaum et al., 2021; Karan et al., 2014). Crowe (2004, p. 327) even states that “BPD may be better described as a chronic shame response.” Similarly, Unoka and Vizin (2017) found that patients with BPD reported more shame and were more prone to angry reactions than other patients without PD and healthy controls, while Rüsch et al. (2007) found more self-reported shame, anger, and hostility in BPD participants compared to participants with social phobia or healthy controls. Hence, research suggests that shame may—at least in part—explain aggressive behaviors in people presenting borderline traits (Peters & Geiger, 2016; Rüsch et al., 2007).

### Narcissistic Rage

Kohut’s theory (1972) on narcissistic rage offers a theoretical framework to understand aggressive behaviors following a provocation in a context of narcissistic vulnerability (Hart et al., 2017). According to Kohut, narcissistic rage is a distinct type of aggression, mixed with anger and destruction, in which aggression defends a grandiose self-overwhelmed by anger, mistrust, and shame (Krizan & Johar, 2015). This rage usually acts as a response to threatened egotism (Kjærvik & Bushman, 2021) or shame (Morrison, 1999). According to this model, shame would be essential for narcissistic rage to happen (Thomaes et al., 2008). A literature review by Lambe et al. (2018) studied the mediating role of narcissism between threatened egotism and aggression. They concluded that narcissism was a relevant variable to understand aggression, and that the prevalence of aggression was stronger following an ego threat. In another study, Krizan and Johar (2015) have examined the relationship between pathological narcissism, shame, and aggression to determine if narcissistic traits were predictors of shame and aggression. Their results show that a high level of narcissistic vulnerability predisposes an individual to aggressive responses when facing a provocation. Indeed, narcissistic vulnerability was associated with physical and verbal aggression, hostility, and anger, whereas narcissistic grandiosity was only linked to physical aggression. In line with these results, narcissistic rage does not appear to be a feature of narcissistic grandiosity but rather of narcissistic vulnerability.

### Aims of the Present Study

The aim of the present study is to investigate, using path models, the interrelations between narcissistic vulnerability, shame, and aggression. These variables correspond to the narcissistic rage framework described by Kohut (1972). More specifically, we aim to determine which model best describes narcissistic rage by comparing a set of competing models inspired by theoretical and empirical literatures. By doing so, we wish to examine the possible mediating role of shame in narcissistic rage. A secondary objective of this study is to determine if narcissistic rage is exclusive to narcissistic vulnerability or if it could also be part of narcissistic grandiosity and borderline traits. Indeed, theory shows that it could be even more pronounced in borderline traits compared with pathological narcissism (Wolf, 1988). To our knowledge, no previous study has tested this assumption.

**Figure 1.**
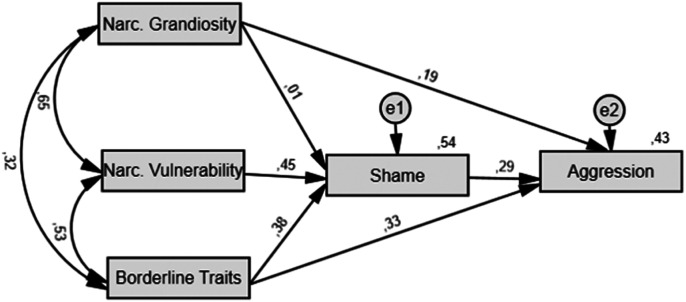
Model A: Path Model Linking Pathological Narcissism and Borderline Traits to Trait Aggression Through Shame. *Note.* Single-headed arrows represent relationships between variables with the standardized path coefficient (β) displayed above, while double-headed arrows show Pearson’s correlations between variables (all *p*s < .05). Numbers written at the top right of a box correspond to the variance explained (*R*^2^) by predictors (all *p*s < .05). Narc. = Narcissistic. e = error or unexplained variance.

**Figure 2.**
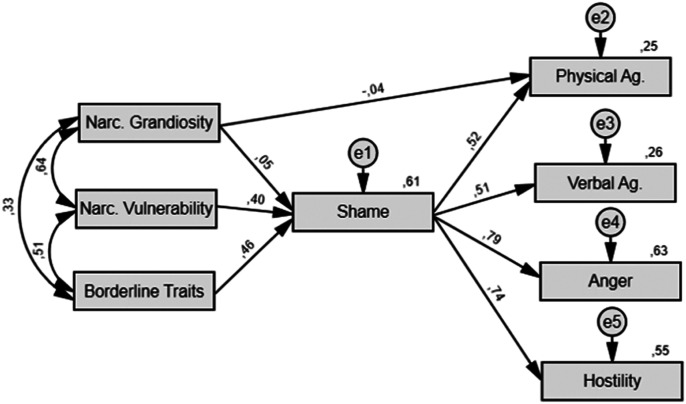
Model B: Path Model Linking Pathological Narcissism and Borderline Traits to Specific Types of Aggression Through Shame. *Note.* Single-headed arrows represent relationships between variables with the standardized path coefficient (β) displayed above, while double-headed arrows show Pearson’s correlations between variables (all *p*s < .05). Numbers written at the top right of a box correspond to the variance explained (*R*^2^) by predictors (all *p*s < .05). Narc. = Narcissistic. e = error or unexplained variance. Ag. = Aggression.

**Figure 3.**
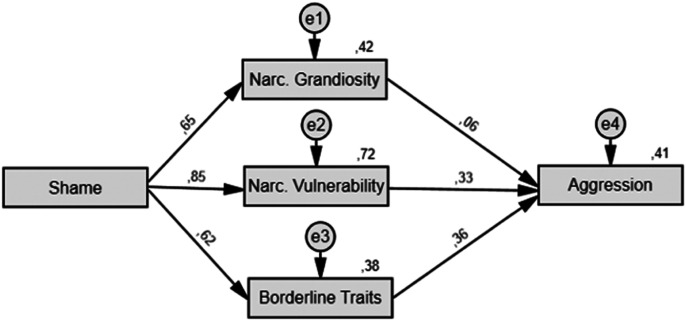
Model C: Path Model Linking Shame to Trait Aggression Through Personality Variables. *Note.* Single-headed arrows represent relationships between variables with the standardized path coefficient (β) displayed above. Numbers written at the top right of a box correspond to the variance explained (*R*^2^) by predictors (all *p*s < .05). Narc. = Narcissistic. e = error or unexplained variance.

Three hypothetical models were tested (see Figures 1–3). Model A links personality variables to shame and then to trait aggression. Narcissistic grandiosity and borderline traits were also directly linked to aggression, in line with some previous studies (e.g., Krizan & Johar, 2015; Mancke et al., 2015). Model B connects personality variables to shame and then to different types of aggression (physical and verbal aggression, anger, and hostility). Narcissistic grandiosity was also directly linked to physical aggression as suggested by Krizan and Johar’s (2015) results. Lastly, Model C tested personality variables as mediators of the narcissistic rage phenomenon as suggested by Lambe et al.’s (2018) literature review. This view of personality variables as mediators, albeit plausible, is different from what is currently assumed in most research articles. Thus, we decided to include it as a third hypothetical model.

We hypothesize that: (a) narcissistic rage can be represented by one of the hypothesized models (see Figures 1–3); (b) considering the associations between narcissistic vulnerability, shame, and aggression found in the literature (e.g., Krizan & Johar, 2015), shame will act as a mediator between narcissistic vulnerability and aggression; (c) narcissistic grandiosity will be linked to physical aggression only (Krizan & Johar, 2015), and shame will not mediate this relationship; and (d) considering the associations between borderline traits, shame, and aggression (e.g., Peters & Geiger, 2016), narcissistic rage can also be associated with borderline traits.

## Method

### Participants and Procedure

A total of 399 French-Canadian participants (192 women [48.1%]; 198 men [49.6%]; 9 participants [2.3%] identified with another gender, or declined to answer) were recruited online via a mailing list destined to researchers, clinicians, and students in the field of psychology in the Province of Quebec, Canada, and on social media via publicity targeting potential adult participants in the province. Participants were aged between 18 and 78 years old (*M*_*age*_ = 40.6; *SD* = 16.3), most were full-time workers (41.9%) or students (27.3%), and most were involved in a romantic relationship (61.1%). All participants gave informed consent to complete a battery of online questionnaires.

### Measures

Narcissistic grandiosity and vulnerability were measured using the brief version of the Pathological Narcissism Inventory (B-PNI; Schoenleber et al., 2015; French validation by Diguer et al., 2020). It is a 28-item dimensional personality questionnaire assessing grandiose (α = 0.78; MacDonald’s Omega [ω] = 0.79)^2^ and vulnerable (α and ω = 0.89) aspects of pathological narcissism. Participants had to rate how each item represents them using a 6-point Likert scale. In line with the purpose of our study, we chose the PNI as it focuses on maladaptive features of narcissism, in contrast with other commonly used measures of trait narcissism such as the Narcissistic Personality Inventory (NPI; Raskin & Hall, 1981) that cover both adaptive and maladaptive components (Ackerman et al., 2011).

Borderline traits were measured using the short form of the Borderline Symptom List (BSL-23; Bohus et al., 2009; French validation by Nicastro et al., 2016), a 23-item dimensional personality questionnaire. Participants were invited to rate the presence of symptoms typical of borderline pathology during the previous week on a 5-point Likert scale. Only the total score was used (α and ω = 0.95).

Shame was measured using the Experience of Shame Scale (ESS; Andrews et al., 2002; French validation by Théberge et al., 2021), a 25-item questionnaire assessing shame proneness. Participants had to rate their shame experiences in a variety of areas of their life (e.g., their personal habits, following a failure, or their body image) on a 4-point Likert scale. Only the total score was used (α and ω = 0.94).

The short version of the Buss and Perry Aggression Questionnaire (BPAQ; Bryant & Smith, 2001; French validation by Genoud & Zimmermann, 2009) was used to measure aggression. It is a self-report questionnaire counting 12 items assessing: (a) Physical aggression (α = 0.78; ω = 0.79); (b) Verbal aggression (α and ω = 0.61); (c) Anger (α and ω = 0.83); and (d) Hostility (α = .72; ω = 0.77). The total score was also computed and interpreted as a trait aggression score (α = 0.85; ω = 0.86). Participants were invited to rate how each item represents them using a 5-point Likert scale.

### Data Analysis

This study compared competing path models to identify what arrangement of variables—and interrelations between them—best describe the data. These models also allowed us to examine direct and indirect effects of exogenous variables (i.e., narcissistic grandiosity and vulnerability, borderline traits) on endogenous variables (i.e., shame and aggression and its subtypes). Model C presents a different arrangement of variables in which shame is the exogenous variable, and personality traits and trait aggression are endogenous variables. Path analysis can be defined as variations of multiple regression analysis allowing the exploration of relationships between variables in a specified model (Stage et al., 2004). They require strong theoretical assumptions as the researcher must decide prior to the analysis which models to test (Garson, 2014). They also require a sufficient sample size; Kline (2016) recommended the following rule of thumb: 10 participants—or ideally 20 participants—per parameter, or at least 200 participants. In our study, following this rule, for the least parsimonious model (Figure 2), 160 participants would have been minimally required to have enough statistical power.

All models were performed on IBM SPSS Amos version 26.0 (Arbuckle, 2019) using the unweighted least squares (ULS) estimation method, as it best fits ordinal data (Xia & Yang, 2019). We also selected a set of fit indices that allowed us to compare the competing models included in this study: The goodness-of-fit (GFI; > 0.95) and adjusted goodness-of-fit (AGFI; > 0.95) indices (Schreiber et al., 2006); the standardized root mean square residual (SRMR; < 0.08; Hu & Bentler, 1999); and the normed fit index (NFI; > 0.95; Schreiber et al., 2006). Lastly, standardized (β) and unstandardized (*B*) path coefficients were estimated using 95% confidence intervals through bootstrapping using 250 bootstrap samples (Nevitt & Hancock, 1998).

## Results

An examination of path models and coefficients (see Figures 1–3) suggested that narcissistic vulnerability and borderline traits are strong predictors of shame which, in turn, predicts trait aggression (Model A) and specific types of aggression (Model B). It appeared that path coefficients for the relationship between narcissistic grandiosity and shame (Models A and B), and between shame and physical aggression, were nonsignificant, as displayed in Table 1. As aforementioned, Model C is different as we tested another arrangement of variables suggested by Lambe et al. (2018), who posit that narcissism could influence the relationship between shame and aggression. Thus, Model C indicated that shame predicts all three personality variables, and that narcissistic vulnerability and borderline traits are predictors of aggression, which was not the case for narcissistic grandiosity. It is of note that correlations between exogenous variables in Models A and B were all positive and ranging from moderate to strong in magnitude (*r* range = 0.32–0.65, *p* < 0.05).

**Table 1.** Estimates (B), Path Coefficients (β; Standardized Estimates), and R^2^ of Competing Path Models.

Model	Endogenous Variable	Exogenous Variable	*B* [95% CI]	β [95% CI]	*R*^2^
A	Shame				0.544*
		Narcissistic grandiosity	0.007 [−0.068–0.098]	0.009 [−0.088–0.123]	
		Narcissistic vulnerability	0.310* [0.210–0.383]	0.454* [0.301–0.562]	
		Borderline traits	0.318* [0.244–0.407]	0.381* [0.291–0.487]	
	Aggression				0.428*
		Narcissistic grandiosity	0.177* [0.085–0.265]	0.190* [0.090–0.292]	
		Shame	0.339* [0.184–0.585]	0.286* [0.158–0.475]	
		Borderline traits	0.324* [0.132–0.448]	0.327* [0.138–0.454]	
B	Shame				0.615*
		Narcissistic grandiosity	0.032 [−0.026–0.099]	0.051 [−0.044–0.152]	
		Narcissistic vulnerability	0.217* [0.142–0.289]	0.403* [0.273–0.513]	
		Borderline traits	0.305* [0.244–0.379]	0.464* [0.378–0.560]	
	Physical aggression				0.253*
		Narcissistic grandiosity	−0.044 [−0.153–0.072]	−0.044 [−0.145–0.072]	
		Shame	0.859* [0.575–1.163]	0.521* [0.400–0.627]	
	Verbal aggression				0.257*
		Shame	0.886* [0.634–1.172]	0.507* [0.389–0.609]	
	Anger				0.627*
		Shame	1.700* [1.339–2.079]	0.792* [0.717–0.854]	
	Hostility				0.546*
		Shame	1.687* [1.458–1.947]	0.739* [0.665–0.800]	
C	Narcissistic grandiosity				0.416*
		Shame	0.929* [0.754–1.132]	0.645* [0.576–0.721]	
	Narcissistic vulnerability				0.718*
		Shame	1.393* [1.201–1.669]	0.847* [0.790–0.895]	
	Borderline traits				0.381*
		Shame	0.833* [0.690–0.968]	0.617* [0.531–0.693]	
	Aggression				0.410*
		Narcissistic grandiosity	0.059 [−0.026–0.144]	0.064 [−0.027–0.156]	
		Narcissistic vulnerability	0.269* [0.188–0.354]	0.332* [0.229–0.429]	
		Borderline traits	0.355* [0.242–0.467]	0.360* [0.252–0.466]	

*Note.* Endogenous variables are the ones predicted by other variables included in the model, while exogenous variables are the ones that are not predicted by variables included in the model. CI = confidence interval.

*p < .05.

Considering *R*^2^ values displayed in Table 1 and Figures 1–3, personality variables in Models A and B, respectively, accounted for 54 and 61% of the variance of shame, while Models A and C, respectively, explained 43 and 41% of the variance of trait aggression. In Model B, we observed that shame appeared to be a better predictor of anger (*R*^2^ = 0.63) and hostility (*R*^2^ = 0.55) than of physical (*R*^2^ = 0.25) or verbal (*R*^2^ = 0.26) aggression, which suggests that our models better capture trait aggression rather than specific aspects of this construct.

An appraisal of fit indices of all three competing models showed that Model A obtained the best fit (GFI = 0.999; AGFI = 0.978; SRMR = 0.024; NFI = 0.997), while Model B (GFI = 0.982; AGFI = 0.961; SRMR = 0.103; NFI = 0.967) and Model C (GFI = 0.989; AGFI = 0.958; SRMR = 0.084; NFI = 0.978) both had some problematic fit indices.

## Discussion

This study aimed to investigate the interrelations between narcissistic vulnerability, shame, and aggression through the scope of narcissistic rage as introduced by Kohut (1972). Kohut’s theory posits that vulnerable narcissistic individuals can react to narcissistic injuries by shameful withdrawal or narcissistic rage, that is, a vengeful aggression aiming to restore a grandiose sense of self following a narcissistic injury that instilled shame in the person. In this study, we wanted to examine if shame had a mediating role in the pathway from narcissistic vulnerability to aggression. We were also interested in exploring, as a secondary aim of the study, if narcissistic rage could also manifest in narcissistic grandiosity and borderline traits.

To test these hypotheses, we resorted to three competing path models including these variables. Model A outperformed the others as shown by the close-to-perfect GFI and NFI, and the SRMR that was much below the usual cut-off value. Model A was also the most parsimonious in including only trait aggression (BPAQ total score). Hence, according to West et al. (2012), it is unsurprising that this model obtained the best fit indices as models with a low number of parameters often perform better. Model B appeared as an appealing model for the detailed information it provides, especially about aggression and its subtypes. More specifically, it shows that shame and narcissistic vulnerability have respectively direct and indirect effects on all kinds of aggression covered by the BPAQ, which is in line with previous studies (Krizan & Johar, 2015; Rasmussen, 2016). Interestingly, Model B shows that the pathway from narcissism to aggression through shame explains more than half of the variance for anger (63%) and hostility (55%). Taken together, these results suggest that the narcissistic rage model could better account for an internalizing kind of aggression (anger and hostility), while predicting more modestly externalizing aggressive behaviors (physical and verbal aggression). This assumption is in line with the centrality of envy—an important component of the Hostility subscale in the BPAQ—and other internalizing, negative emotions found in narcissistic vulnerability (Kealy & Rasmussen, 2012; Krizan & Johar, 2012). Narcissistic grandiosity, which was not associated with narcissistic rage, is more consistently associated with overt grandiosity and exhibitionistic behaviors (Pincus & Lukowitsky, 2010). Model C tested Lambe et al.’s (2018) view of personality variables as mediators between shame and aggression. This model was included in this study to test a different arrangement of variables supported by the literature, allowing us to confront our original hypothesis regarding the order of entry of the variables in the model. Two reasons led us to reject it: (a) the theory behind this model is not as sound as for the other models whereas path models should rely on sound theory to be considered valid (Garson, 2014; Stage et al., 2004) and (b) the SRMR is above the cut-off value. We consider this arrangement of variables as atypical as most research in the fields of aggression (e.g., Anderson & Bushman, 2002; Kjærvik & Bushman, 2021) and personality (e.g., Freis et al., 2015; Thomaes et al., 2008) posits that shame acts as a predictor in the pathway from pathological personality traits to aggression. One main difference between the present study and Lambe et al.’s work that could account for the discrepancy in our results lies in their unitary conceptualization of pathological narcissism (i.e., not distinguishing between grandiose and vulnerable dimensions). This conceptualization of pathological narcissism is increasingly seen as problematic as it does not account for vulnerable aspects (e.g., depleted self-image and feelings of despair and emptiness), but often solely relies on overt grandiosity (Pincus et al., 2014).

Moreover, results from Models A and B suggest that shame acts as a mediator between narcissistic vulnerability and aggression. This confirms previous findings, for instance, from Thomaes et al. (2008) whose study investigated shame-induced aggression in teenagers. After participants were told they had lost in a game against a supposedly bad player, teenagers could blast their opponent with noise. Participants higher in narcissism showed more aggression in contrast with healthy controls, but only after they had felt shame. Our models also confirm results from a more recent study (Hart et al., 2017) that suggested that narcissistic vulnerability displays all features of narcissistic rage by its association with shame, aggression, and hostility. To our knowledge, only one study reported discrepant findings. Fjermestad-Noll et al. (2020) studied a population of depressed individuals with and without narcissistic PD. On the contrary, they found that shame reduces aggression in their sample when participants had perfectionistic traits; this result could be partly explained based on Schoenleber and Berenbaum’s (2012) proposition that perfectionism, that is, the tendency to maintain high standards or to avoid the exposure of personal flaws, can act as a preventive strategy to avoid or regulate shame in narcissism, possibly reducing the use of aggression as a regulatory strategy. Notwithstanding the inclusion of similar variables in our respective studies, Fjermestad-Noll et al. (2020) studied a very specific clinical sample that limits comparability with ours. Overall, our results support the idea conveyed within the narcissistic rage literature that key features of this phenomenon (i.e., shame, anger, and aggression) are found in narcissistic vulnerability, which would consequently be the strongest predictor of narcissistic rage when compared with narcissistic grandiosity (Hart et al., 2017; Krizan & Johar, 2015).

The lack of association between narcissistic grandiosity and shame suggests that the former has questionable relevance to the study of narcissistic rage. This is in line with the fact that shame is not theoretically expected to be a core component of narcissistic grandiosity (Kealy & Rasmussen, 2012), although some studies mitigated this assumption by reporting weak associations between the two (e.g., Di Sarno et al., 2020). Theory (e.g., Tracy et al., 2011) and research (e.g., Uji et al., 2012) show that individuals high on the grandiose side of pathological narcissism tend to shield from shame with hubris, externalization of painful feelings, and overt grandiosity.

Contrary to our expectations, our results do not confirm the assumption from Krizan and Johar (2015) that narcissistic grandiosity is linked to physical aggression as, in Model B, there was no relationship between the two. This unexpected result may be due to the chosen measure to assess narcissistic grandiosity; Krizan and Johar used the NPI (Raskin & Hall, 1981), while we opted for the narcissistic grandiosity scale of the PNI. This scale includes a subscale covering “Self-Sacrificing Self-Enhancement,” which entails sacrificing oneself with the objective of improving one’s self-image, which is hardly compatible with physical aggression (i.e., a participant would be unlikely to endorse both). Direct effects between narcissistic grandiosity and other types of aggression (verbal aggression, hostility, and anger) were not included in the model for the sake of parsimony; furthermore, we had no empirical rationale to do so, as Krizan and Johar found no significant positive relationship between these variables. Once again, Model A seems more in line with previous literature as it displays positive associations between narcissistic grandiosity and trait aggression but not in reaction to shame. Further studies should focus on developing a better understanding of aggressive tendencies in narcissistic grandiosity. It remains possible that the relationship between the two could be mediated by other variables not included in the present study such as impulsivity and spitefulness (Rogier et al., 2019), or self-control (Rasmussen, 2016).

Our results also support the assumption that narcissistic rage is associated with higher borderline traits, as theoretically suggested by Wolf (1988). More recent works (e.g., Peters & Geiger, 2016) also pointed out that shame and aggression are major features of borderline traits. Our results indicate that two different paths—direct and indirect—can lead to aggression in borderline traits. This suggests that, in borderline traits, shame can lead to reactive aggression, but that there is also a direct association between the two. Such a conclusion entails that aggressive behaviors in borderline traits are complex and may be prompted by several factors including, but not limited to, shame. Future studies should include impulsivity and emotion dysregulation, as suggested by Mancke et al. (2015), in addition to shame as potential mediators to better understand aggression in borderline traits. This study is a first step in the empirical study of narcissistic rage in borderline traits, and further research is required to better describe this phenomenon in this specific context.

One important caveat to our results lies in the definition of shame that is highly influenced by culture. Indeed, several differences have been observed regarding the definition and the connotation of shame between Eastern and Western cultures (Wong & Tsai, 2007). In Western cultures, shame is viewed mostly as a painful, maladaptive emotion (Collardeau et al., 2021) whereas shame is a socially prescribed emotion in collectivistic cultures (often Eastern societies) as the negative evaluation and the shame that ensues are viewed as sources of information and motivation for self-improvement (Wong & Tsai, 2007). To illustrate this phenomenon, Pakistani immigrants in Canada were interviewed in a recent study (Collardeau et al., 2021). The participants reported a nuanced understanding of shame. For the most part, it was conceptualized as a motivation to improve oneself, mostly leading to coping strategies such as sharing with others or accepting and correcting the perceived wrongdoing. Negative regulation strategies such as withdrawal were also reported at times. These observations cast doubt on the generalization of the present results to Eastern or collectivistic cultures given the prominence of positive shame regulation strategies. A recent meta-analytic review (Kjærvik & Bushman, 2021) found that the association between narcissism and aggression was not influenced by the individualistic or collectivistic culture of the participants. However, as there were only 12% of the 437 studies included that recruited participants from a collectivistic culture, we can still wonder if the definition of shame endorsed by a given culture is likely to have a significant impact on how to understand causes of aggression across cultures and countries. Hence, future research including participants from a collectivistic culture is warranted.

### Limitations

Some limitations and diversity issues regarding the present findings must be addressed. First, our participants were all French-Canadian individuals; thus, this sample may not be representative of the diverse North American population. The lack of data on the ethnic background or sexual diversity of participants, most notably, limits the generalization of our results to more diverse population groups. In their current form, results may not necessarily apply to Black, Indigenous and People of Color (BIPOC) populations, and further research involving individuals belonging to a vast array of cultural groups is warranted, especially given the importance of diversity and equity in the field of interpersonal violence (e.g., Bent-Goodley, 2021). Moreover, this study resorted to self-report questionnaires to assess personality traits, shame, and aggressive behaviors in participants; thus, it is correlational in nature. Future studies involving experimental designs are warranted to expand the knowledge on the associations between personality, shame, and aggression. An important caveat regarding path modeling is that although they were used to examine the influence of a variable on another, they cannot establish causality between variables, or which model should be retained over another (Garson, 2014). The ESS assesses shame proneness, and implicit measures of shame could enhance ecological validity in capturing state shame that may not always be disclosed by participants. In addition, as this study is the first to our knowledge to test models representing the narcissistic rage, we wanted to test simple and parsimonious models. We are aware that some covariates (e.g., age and gender) could have been included; however, Kjærvik and Bushman (2021) found that no such covariates significantly affected the associations between narcissism and aggression. Future studies should nevertheless pay attention to the role of these covariates while testing more complex models including other potential mediators (e.g., impulsivity and emotion regulation). Lastly, we studied a community sample in which levels of pathology were relatively low; for instance, only 7.02% of participants reported “high to extremely high” borderline symptoms according to Kleindienst et al.’s (2020) BSL-23 classification of severity levels^3^. Personality interviews or recruiting participants in outpatient clinics could favor an increase in pathology level in further studies.

## Conclusion

The aim of this study was to investigate the interrelations between pathological narcissism, borderline traits, shame, and aggression in line with Kohut’s (1972) theory of narcissistic rage. Using path models, narcissistic vulnerability and borderline traits showed positive associations with shame and aggression, while shame acted as a mediator between the aforementioned personality traits and aggression. On the opposite, narcissistic grandiosity only showed a direct effect on aggression. Four important conclusions can be drawn from our study: (a) our findings add evidence in favor of the conceptualization of narcissistic rage introduced by Kohut (1972); (b) they also stress the centrality of shame in the relationship between narcissistic vulnerability, borderline traits, and aggression; (c) our findings suggest that narcissistic rage could better account for internalizing types of aggression such as anger and hostility rather than externalizing manifestations of aggression (e.g., verbal and physical); and (d) narcissistic rage may not be exclusive to narcissistic vulnerability, but could also manifest in borderline traits. Hence, future research is warranted to expand the knowledge on narcissistic rage’s characteristics, conceptualization, and measurement. In addition, previous studies (e.g., Rasmussen, 2016; Rogier et al., 2019) identified other variables (i.e., impulsivity, spitefulness, and self-control) that could potentially act as mediators between narcissistic vulnerability and aggression. Future research should focus on such variables and compare their respective role in the narcissistic vulnerability-aggression interaction to better understand what leads to aggression in pathological narcissism. Lastly, the present study did not include self-aggression as a type of aggression; future studies should focus on this specific type of aggression as pathological narcissism and borderline traits have been previously linked to shame-based suicide and self-harm (e.g., Bohus et al., 2021; Links, 2013).

## Notes

1. We use “shame” and “threatened egotism” interchangeably as the two concepts largely overlap, as the latter can be defined as an event that challenges the person’s positive self-view (Leary et al., 2009).

2. Cronbach’s alphas and MacDonald’s omegas were calculated using JASP software version 0.14.1.0 (JASP Team, 2020).

3. To our knowledge, no such classification of symptoms exists for the PNI to this day.
